# Identification of functional enhancer variants associated with type I diabetes in CD4+ T cells

**DOI:** 10.3389/fimmu.2024.1387253

**Published:** 2024-06-14

**Authors:** Arpit Mishra, Ajay Jajodia, Eryn Weston, Naresh Doni Jayavelu, Mariana Garcia, Daniel Hossack, R. David Hawkins

**Affiliations:** ^1^Division of Medical Genetics, Department of Medicine, University of Washington School of Medicine, Seattle, WA, United States; ^2^Department of Genome Sciences, University of Washington School of Medicine, Seattle, WA, United States; ^3^Institute for Stem Cell and Regenerative Medicine, University of Washington School of Medicine, Seattle, WA, United States; ^4^Benaroya Research Institute at Virginia Mason, Seattle, WA, United States

**Keywords:** type 1 diabetes, non-coding variants, enhancer elements, GWAS, 3D genome architecture, massively parallel reporter assay (MPRA)

## Abstract

Type I diabetes is an autoimmune disease mediated by T-cell destruction of β cells in pancreatic islets. Currently, there is no known cure, and treatment consists of daily insulin injections. Genome-wide association studies and twin studies have indicated a strong genetic heritability for type I diabetes and implicated several genes. As most strongly associated variants are noncoding, there is still a lack of identification of functional and, therefore, likely causal variants. Given that many of these genetic variants reside in enhancer elements, we have tested 121 CD4+ T-cell enhancer variants associated with T1D. We found four to be functional through massively parallel reporter assays. Three of the enhancer variants weaken activity, while the fourth strengthens activity. We link these to their cognate genes using 3D genome architecture or eQTL data and validate them using CRISPR editing. Validated target genes include *CLEC16A* and *SOCS1.* While these genes have been previously implicated in type 1 diabetes and other autoimmune diseases, we show that enhancers controlling their expression harbor functional variants. These variants, therefore, may act as causal type 1 diabetic variants.

## Introduction

Type I diabetes mellitus (T1D) is categorized as an autoimmune disease caused by T-cell-mediated loss of β cells in pancreatic islets of Langerhans that leads to insulin deficiency. Autoreactive T cells are known to be key mediators of β-cell destruction (1). T1D is most commonly present in childhood or adolescence; however, it can appear at any age. The genetic factor comprises ~80% of an individual’s risk for developing the disease. In the past decade, a series of genome-wide association studies (GWAS) have identified more than 50 genetic loci for conferring T1D risk (2–4). As most of the underlying single nucleotide polymorphisms (SNPs) associated with T1D, as well as those in linkage disequilibrium (LD), are in noncoding sequences, interpreting their role in T1D is challenging.

The development of massively parallel reporter assays (MPRAs) has tremendously increased our capacity to test regulatory sequences carrying genetic variation for any potential effect (5, 6). To determine differences in allelic enhancer activity, MPRA can be utilized to test thousands of regulatory alleles in a single reporter library (7). Previously, it had been applied to characterize cancer-associated variants in cancerous cell lines, blood cell disease traits, and heart disease-associated variants (8–10).

In the current study, we employ MPRAs to quantify the relative effect of T1D variants located in regulatory sequences identified in activated CD4+ T cells. We tested 121 enhancers and 242 alleles, of which four variants showed significant differential enhancer activity. Three of these variants are in the *CLEC16A* gene locus, and one lies in the *BCL2L15* gene locus. Target genes for these functionally validated T1D enhancers are assigned by promoter capture Hi-C and expression quantitative trait loci (eQTL), a subset of which we validate using CRISPR inhibition (CRISPRi) enhancer repression.

## Methods

### Prioritization and functional analysis of variants

We downloaded the NHGRI GWAS Catalog (https://www.ebi.ac.uk/gwas/docs/file-downloads), and all coordinates are hg19. We identified 11 T1D trait SNPs associated (*p* < 1.0 × 10^−5^) with the disease, respectively. Next, we identified proxy SNPs in linkage disequilibrium (LD) with a GWAS SNP based on the 1,000 Genomes Project of CEU ancestry by utilizing the SNAP web server (http://archive.broadinstitute.org/mpg/snap/ldsearch.php). We overlapped the set of T1D-associated SNPs with our previously described H3K4me1 ChIP-seq data, a putative enhancer mark, from primary human CD4+ T cells (11) and identified 121 noncoding variants in enhancer regions.

### Design and synthesis of MPRA library

For the multiplex enhancer SNP validation assay, we leveraged synthetic oligonucleotide array synthesis and adopted self-transcribing active regulatory region sequencing (STARR-seq) (12) (https://www.addgene.org/71509/). An oligonucleotide library was synthesized containing the 230 nucleotide genomic regions as previously described (13).

### Activated CD4+ T-cell culture

MPRA experiments were performed in activated CD4+ T cells. Human CD4+ T cells were obtained from Precision for Medicine, grown at 37°C and 5% CO_2_ in TexMACSTM (Miltenyi-Biotech, Gaithersburg, MD, USA) medium with 10% human serum (Sigma, Saint Louis, MO, USA) and 1% penicillin–streptomycin (Gibco, Grand Island, NY, USA). Naive CD4+ T cells were activated with bead-bound anti-CD3-biotin and anti-CD28-biotin, and expansion of activated CD4+ cells was carried out by adding IL-2 at 10 ng/mL (Miltenyi Systems) to the cultures at 48 h. Cells were electroporated with a Neon transfection kit and device (Invitrogen, Bend, OR, USA) with the following transfection parameters—pulse voltage: 2,100, pulse width: 20, and pulse number: 1.

### MPRA output library construction

MPRA library preparation was performed as previously described (13). Briefly, after cell collection, total RNA from rinsed cell pellets was prepared using the QIAGEN RNeasy kit. Poly-A RNA was isolated from 50 μg of total RNA by µMACS mRNA isolation kit (Miltenyi Biotech). RNA was then treated with turboDNase (4 U) for 30 min at 37°C (Invitrogen). DNase-treated poly-A RNA was purified using the RNeasy kit. Plasmid-specific cDNA was synthesized using Superscript III (Life Technologies, Austin, TX, USA) incubated for 1.5 h at 55°C and inactivated at 80°C for 15 min. Following synthesis, cDNA was treated with RNaseA (Sigma) at 37°C for 30 min. cDNA was purified using AMPure beads in a 1.5:1 bead:cDNA ratio and then amplified and indexed for sequencing using a two-stage PCR as described previously ((12). The cDNA sample from each replicate was used as an input into first-round gene-specific PCR reaction, and KAPA hi-fidelity polymerase (KAPA Biosystem, Wilmington, MA, USA). All libraries were sequenced on the NextSeq 550 (Illumina) performing 1 × 75 cycles.

### MPRA data normalization and analysis

Sequencing raw reads from RNA and plasmid libraries are checked for adapter sequences and low-quality reads (*q* score < 20) using FASTQC (https://www.bioinformatics.babraham.ac.uk/projects/fastqc/) and trimmed using Trim Galore (https://www.bioinformatics.babraham.ac.uk/projects/trim_galore/). Trimmed reads were mapped to the amplicon library using the Bowtie2 aligner (14). The mapped reads were quantified against all tested sequences using the “featureCounts” function from the Rsubread package (15). We employed quantitative allele-specific analysis of reads (QuASAR)-MPRA (16) to define allele-specific activity from MPRA read counts. Allelic enhancer activity *p*-values are adjusted with the Benjamini–Hochberg method to control for multiple testing. Significant allelic enhancer activity was defined at an FDR < 10%.

### T1D enhancer SNP transcription factor binding site analyses

We utilized motifbreakR in R to determine the effect of significant enhancer variants from MPRA on TF binding (19). motifbreakR uses the position weight matrices (PWM) from multiple databases such as Hocomocos, Jaspar, SwissRegulon, CisBP, and hPDI. motifbreakR calculates a *p*-value for alternate and reference allele versions of the TF PWM and then determines binding difference and significance. For the final results, we only use significant binding changes with a *strong* effect predicted and filtered TFs for expression in activated CD4+ T cells using the DICE database (17) (**Supplementary Table S2**).

### CRISPR inhibition

Guide RNAs (gRNAs) targeting enhancers were designed with Benchling (https://benchling.com) using a 200-bp window around the enhancer variant (**Supplementary Table S3**). The two best-scoring gRNAs were synthesized from Synthego, Redwood City, CA, USA. Reconstituted gRNAs were pooled for each enhancer and electroporated into Jurkat-CRISPRi cells (see below). Nontargeting control (NTC) guides as negative controls were obtained from ThermoFisher, Bend, OR, USA (TrueGuide™ sgRNA Negative Control, nontargeting 1, Catalog No. A35526).

Jurkat cells were transduced with the CRISPRi Lentivirus hEF1a-Blast-dCas9-SALL1-SDS3 from Horizon (Catalog ID: VCAS12247). Cells were cultured in RPMI 1640 with 10% FBS and 15 µg/mL of blasticidin for 2 weeks. Single-cell clones were isolated postflow sorting. Single clones were expanded in the presence of antibiotics and confirmed for Cas9 expression using qPCR. These single-cell clones were cryopreserved. Cryopreserved Jurkat CRISPRi cells were initially cultured in RPMI 1640 supplemented with 10% FBS, 1× normocin (InvivoGen). After 3 days of culture, 15 µg/mL blasticidin was added to the culture medium. Cells were maintained and fed every other day. Cells were collected at a density of 2 × 10^6^ per CRISPRi target or NTC, washed with PBS, and resuspended in 45 µL of buffer T (Neon Transfection Kit). Two 15-µM gRNAs per target enhancer were combined at a 1:1 ratio to a final volume of 6 µL. In total, 5 µL of combined gRNA was then added to a tube of cells and mixed well for each CRISPRi-targeted enhancer. The NTC gRNA was diluted to 15 µM, according to the ThermoFisher protocol. Next, cells were mixed with 5 µL of NTC gRNA in the same manner as the enhancer targets. Using the Neon Electroporation system, 10 µL of cell/gRNA suspension was electroporated at 1,350 V, 10 ms, for three pulses. Cells were placed in 190 µL of rescue medium in a flat bottom 96-well plate and allowed to culture for 3 days postelectroporation. Cells were then removed from the plate, washed with PBS, and frozen until RNA isolation was performed.

RNA was isolated using the Qiagen RNeasy Mini kit with DNase treatment. RNA was eluted in 30 µL of RNase-free water. Concentration was tested using the Qubit RNA High Sensitivity assay. After concentration was tested, cDNA was synthesized using the iScript cDNA kit, using 75 ng as a standard following the manufacturer’s protocol. Post-cDNA synthesis, qPCR was performed using TaqMan probes (ThermoFisher) for pcHi-C/eQTL target genes and *TBP* as the internal control gene (**Supplementary Table S3**). Analysis of qPCR data from targeted enhancers and NTC was performed using the TaqMan delta-delta Ct method in Excel and plotted in GraphPad Prism.

### Statistical tests and visualizations

Any additional statistical tests were performed in the R environment (https://www.r-project.org/). Additional graphs and figures were prepared using R packages, GraphPad Prism, BioRender.com, and Abode Illustrator.

## Result

### Differential regulatory activity of T1D enhancer variants

**Figure 1:**
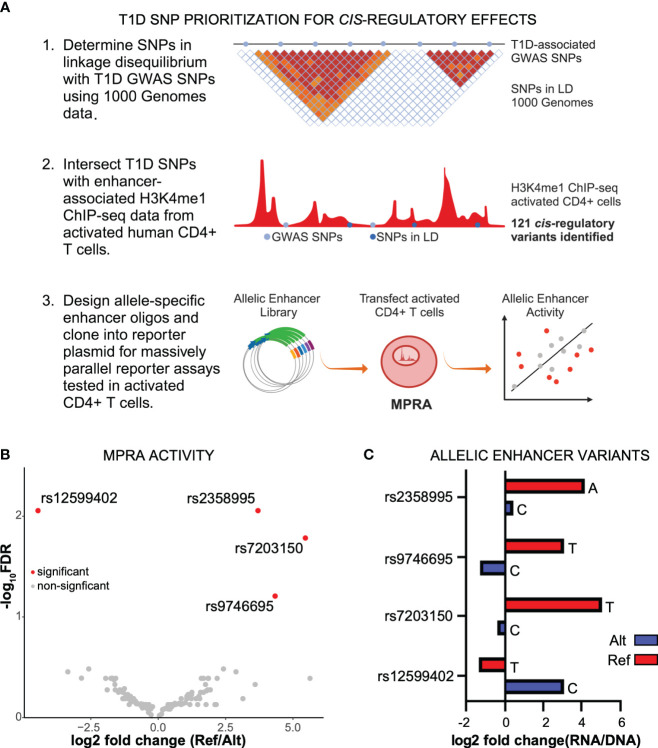
Identification of functional noncoding T1D variants. **(A)** Identification of T1D enhancer variants from activated CD4+ T cells and MPRA design. **(B)** Scatter plot of reference versus alternate MPRA allelic activity for enhancer variants. **(C)** Bar plot of MPRA activity for each significant enhancer allele.

For non-coding variants associated with diseases, such as T1D, to be causal, the variants must have a functional effect. To determine if noncoding, T1D-associated variants have a gene regulatory effect, we first identified SNPs in linkage disequilibrium using 1,000 Genomes data, and then determined if they reside at candidate enhancer elements in activated CD4+ T cells using previously generated chromatin maps of H3K4me1 (11) (**Figure 1A**), which is known to mark enhancers (18). We identified 121 T1D regulatory variants (**Supplementary Table S1**). To determine an allelic difference in enhancer activity due to the T1D variant, the 121 variants (242 alleles) were tested by MPRA in activated CD4+ T cells (**Figure 1A**). After adjusting for multiple corrections with Benjamini–Hochberg (BH) at a 10% False discovery rate (FDR), we were able to identify four variants having differential allelic enhancer activity in activated CD4+ T cells (**Figures 1B, C**). Of these, three variants showed stronger activity for the reference allele, indicating that the alternate allele is weakening enhancer activity, while rs12599402 has stronger activity for the alternate allele (**Figure 1**). Three significant MPRA variants (rs12599402, rs7203150, and rs9746695) are from the *CLEC16A* locus at chromosome 16p13.13 and lie within intronic enhancers within the gene. The fourth variant, rs2358995, is an intronic enhancer variant in the *BCL2L15* and *AP4B1-AS1* genes, an antisense lncRNA on the opposite strand at chromosome 1p13.2. Collectively, these data show that T1D-associated noncoding variants can act in a functional manner to alter enhancer activity.

### Transcription factor binding analysis of functional enhancer variants

**Figure 2:**
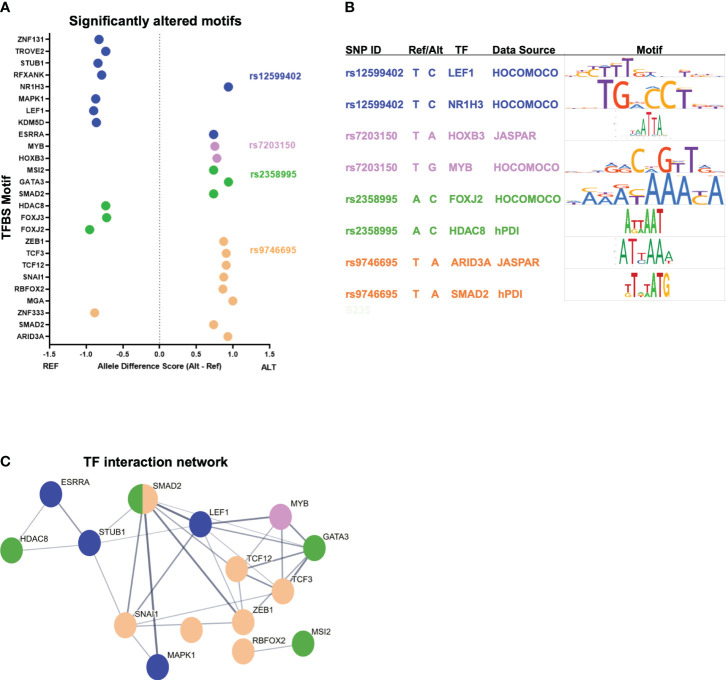
T1D enhancer SNP-associated transcription factor network. **(A)** Top-scoring TF motifs altered by the T1D enhancer variants. **(B)** Example motifs for each variant shown. **(C)** Protein–protein interactions for TFs associated with each T1D enhancer variant. The hub colors correspond to the variant colors in **(A, B)**.

The most likely mechanism for enhancer variants to have a functional effect is by altering transcription factor (TF) binding. We used motifbreakR to predict if each variant is likely to alter TF binding based on the alternate allele change to the corresponding motif (19). After filtering motifs for TF expression in activated CD4+ T cells, each variant is predicted to alter multiple motifs (**Figures 2A, B**; **Supplementary Table S2**). For example, rs9746695 alters motifs for ZEB1, SMAD2, and TCFs. rs12599402 is predicted to change the binding of several factors, including ESRRA, RFXANK, a regulator of HLA class II promoters, and LEF1. The rs2358995 variant impacts SMAD2, GATA3, and FOXJ motifs, whereas rs7203150 is associated with changes in HOXB3 and MYB motifs. Many of these TFs, such as TCF12, LEF1, SMAD2, and GATA3, are known to regulate *SOCS1* and *CLEC16A* by also binding their promoters (20). The homotypic binding of TFs at a gene’s promoter and enhancers is a common feature of gene regulation (21, 22), indicating the above factors may be key regulators of *SOCS1* and *CLEC16A* expression.

To understand how such diverse TFs could play a role in T1D, we asked if there were known relationships between these factors. Protein–protein interactions indicate that many of these TFs do interact (**Figure 2C**). The implication is that individual variants in different enhancers may impact the same complex or similar complexes of TFs by altering the binding of interacting TFs. Furthermore, KEGG pathway analysis of the TFs associated with the functional variants indicates that these factors play a coordinated role in regulating “adherens junction”, “Th17 differentiation”, “human T-cell leukemia virus 1 infection”, and others (**Supplementary Figure S1A**).

### T1D enhancer variant target gene identification

**Figure 3:**
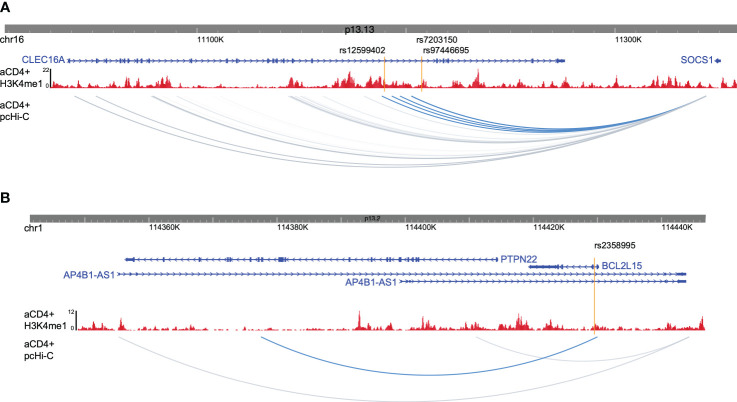
Promoter captures Hi-C enhancer target genes. **(A)** Browser shot of *CLEC16A-SOCS1* locus showing enhancer variants (orange lines), H3K4me1 ChIP-seq data from activated CD4+ T cells (red), and activated CD4+ T-cell pcHi-C data with variant interactions in blue. **(B)** Browser shot of *BCL2L15* locus with data as described in **(A)**.

To gain insight into how enhancer variants contribute to T1D etiology or pathogenesis, we utilized promoter capture Hi-C data from activated CD4+ T cells to identify target genes for each enhancer variant (23), as well as existing eQTL data. rs9746695 and rs7203150 enhancer variants are known eQTL variants for *CLEC16A* expression (Open Targets database; 24). After filtering for expression in activated CD4+ T cells of promoter-capture Hi-C data-interacting genes, the rs12599402, rs9746695, and rs7203150 intronic enhancers of *CLEC16A*, C-type lectin domain containing 16A—a known T1D susceptibility gene (25), have long-range distal interactions over 250 kb with the *SOCS1* and *RMI2* promoters (**Figure 3A**; **Supplementary Figure S1B**). SOCS1, a suppressor of cytokine signaling 1, acts as a negative regulator of interleukin-2 (IL-2), interleukin-3 (IL-3), and interferon-gamma (IFN-γ) signaling. RMI2, RecQ-mediated genome instability 2, is essential for genome stability (26). rs9746695 is an eQTL variant for *RMI2* expression in whole blood (Open Targets database). The *RMI2* isoform, *RMI2-004*, was reported as a T1D risk gene. *RMI* has been shown to gain chromatin accessibility and higher transcriptional output in response to IFN-α expression in islets of T1D individuals (27). All three variants are also eQTL for *DEXI* (Open Targets database), the promoter of which is next to *CLEC16A*. *DEXI*, *a* dexamethasone-induced protein, may play a role in controlling inflammatory responses (28). The rs2358995 variant within the *BCL2L15*, B-cell lymphoma 2-like 15, gene is a previously reported eQTL for *BCL2L15* expression in LCL (29) and transverse colon (30), and for *PTPN22.* The enhancer also shows an interaction with the *PTPN22* gene body, supporting the idea that it may also regulate *PTPN22*, but the variant is too close to the *PTPN22* promoter to distinguish a significant interaction with the promoter from a false positive proximity ligation (**Figure 3B**). *PTPN22* encodes a tyrosine phosphatase that functions as a key regulator of immune homeostasis through regulations of TCR and BCR signaling.

### CRISPR validation of target gene interactions and enhancer activity

**Figure 4:**
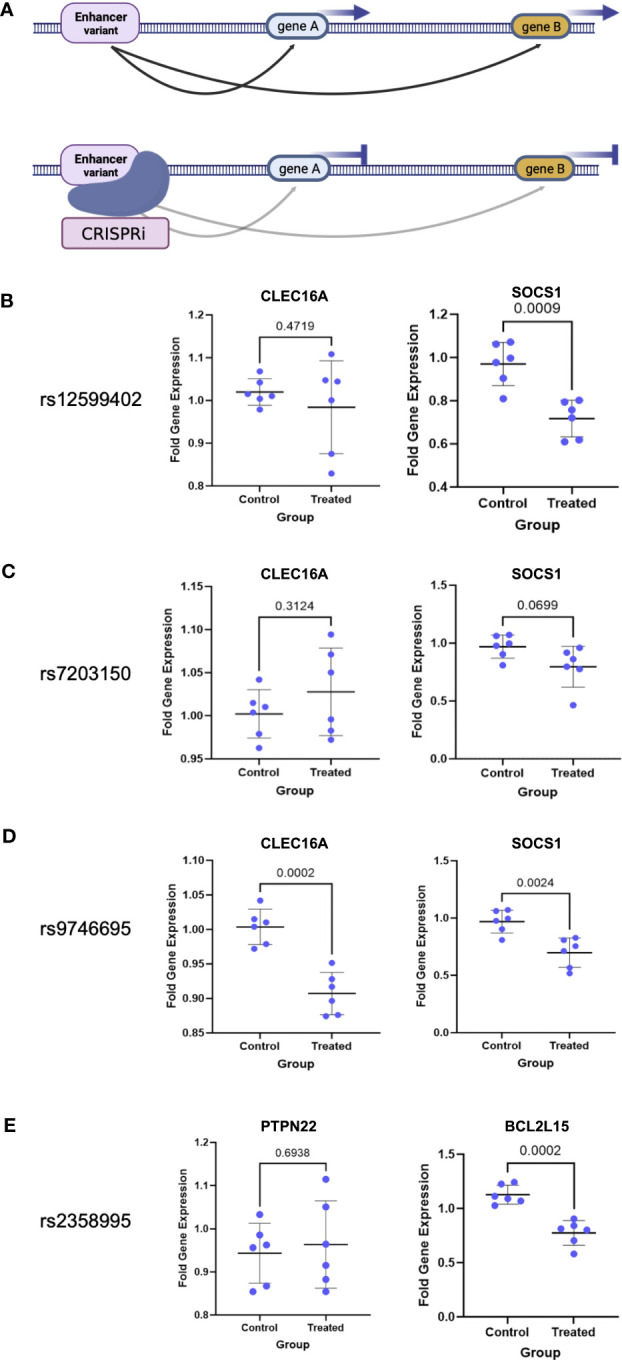
CRISPRi validation of enhancer–target gene interactions. **(A)** Schematic of enhancer interaction and CRISPRi effect on a true target gene. **(B–E)** Enhancer target gene expression level after enhancer (treated) repression by CRISPRi or using nontargeting guides (control). *p*-values are indicated above the line in each comparison.

To confirm the target gene interactions as well as enhancer activity of the region, we established a dead Cas9 (dCas9) CRISPRi Jurkat T-cell line and targeted the CD4+ T-cell enhancers, which are also open chromatin regions in Jurkat cells (**Figure 4A**). We tested the effects of enhancer repression on target expression using qPCR as compared to nontargeting control (NTC) guides. We targeted the three *CLEC16A* intronic enhancer variant regions and tested the effects on *CLEC16A* and *SOCS1* expression. We found significant downregulation of *SOCS1* associated with editing in the rs12599402 (pval = 0.0009) and rs9746695 (pval = 0.0024) enhancers. While the rs7203150 enhancer repression was not significant (pval = 0.0699), *SOCS1* expression trended downward (**Figures 4B, C****).** A significant decrease in expression of *CLEC16A* was only associated with editing the rs9746695 enhancer (pval = 0.0002) (**Figure 4D**). All three variants significantly downregulate *DEXI* expression (**Supplementary Figure S2**). We next targeted the rs2358995 enhancer and tested the effect on *BCL2L15* and *PTPN22. BCL2L15* was significantly downregulated (pval = 0.0002), but no significant change was found for *PTPN22* (pval = 0.6938) (**Figure 4E**).

## Discussion

Genome-wide association studies have proven to be a compelling approach to identifying a genetic basis for T1D, as well as many common human diseases and traits; however, few genes have been found to be causal, although T1D-associated coding variants have been found for several genes (3, 31, 32). As most significant variants lie in a noncoding region of the genome, epigenomic technologies to identify candidate *cis-*regulatory elements, such as enhancers, have breathed new life into GWAS data. SNPs identified from GWAS can affect the function of transcriptional enhancers by altering transcription factor binding and, in turn, target gene expression levels through interactions with distant gene promoters via looping in 3D space. This warrants a critical examination of the genetic variation in *cis-*regulatory elements and a further need to determine their target genes if we are to understand the phenotypic consequences of functional regulatory variants. Such an understanding of variants associated with T1D may provide better insights into the etiology and potentiate novel therapeutic interventions.

In recent years, massively parallel reporter assays have become a powerful approach to dissecting the functional effects of noncoding variants associated with various diseases (7, 33–37). Our MPRA study of T1D-associated variants has pinpointed four enhancer variants from the *CLEC16A* and *BCL2L15* loci as potentially causal. However, these enhancer variants do not necessarily regulate just the nearest neighboring gene and can regulate more than a single gene.

The rs9746695 (chr16:11207894, T/C) and rs7203150 (chr16:11207722, T/C) intronic enhancer variants of *CLEC16A* are known eQTL variants for *CLEC16A* expression, and promoter capture Hi-C indicates interactions with *SOCS1* and *RMI2*. We tested the effect of this enhancer on the expression of *CLEC16A* and *SOCS1* by CRISPRi and found that targeting the rs9746695 region of the enhancer reduced the expression of each gene, but targeting the rs7203150 region of the enhancer had no effect. It is unclear how variants within the same enhancer could have such differing results, other than blocking distinctly different TF-binding sites. One possibility is inefficient targeting by rs7203150 gRNAs. Other technical aspects such as transfection efficiency, could play a role, as not all cells take up the gRNA, therefore, all gene expression is measured in a background of unedited cells. rs9746695 is also a known eQTL variant controlling *RMI2* expression. This indicates that the enhancer controls the expression of at least three genes: *CLEC16A*, *SOCS1*, and *RMI2*. rs12599402 (chr16:11189888, T/C) in an intronic enhancer ~18 kb away also interacts with *SOCS1* and *RMI2* but is not a known *CLEC16A* eQTL variant. Our CRISPRi validation confirmed enhancer activity and control of *SOCS1* expression, but it does not appear to control *CLEC16A. RMI2* was not tested. All three variants are also eQTL variants for *DEXI*, and our CRISPRi experiments confirm that the enhancers are controlling its expression. These functional variants, therefore, can impact the expression of multiple genes, and the expression of a single gene may be impacted by multiple T1D risk variants and distinct enhancer elements.

*CLEC16A* has been identified as a susceptibility gene for type 1 diabetes, multiple sclerosis, and adrenal dysfunction, with a clear role in T cells yet to be determined. *CLEC16A* is a membrane-associated endosomal protein and one of the important regulators of mitophagy. Its role in the pancreas is better studied; however, the function of mitophagy and related pathways in T1D biology is limited (38). A role in autophagy in T-cell survival and proliferation has been linked to the development of autoimmunity in related autoimmune disorders (39–42).

Similar to *CLEC16A*, *DEXI* has also been implicated as a T1D gene. However, recent studies in NOD mice found that *DEXI* knockout mice did not have an increased susceptibility to diabetes in the way that *CLEC16A* knockdown mice did (43).

The *SOCS1* gene has been implicated in multiple autoimmune disorders, though variants in the gene have not been identified as causal. SOCS1 is a suppressor of cytokine signaling, and as such, regulates multiple cytokines involved in immune response, including IL-2 and IFN-γ. SOCS1 operates through the inhibition of the catalytic activity of JAK/STAT pathway members and TYK2. This in turn restricts the overall cellular communications that occur via cytokine signaling. Many of the cytokines regulated by SOCS1 are implicated in inflammation and inflammatory responses (44). Therefore, misregulation of *SOCS1* via enhancer variants may promote a proinflammatory response. Such a proinflammatory response may prove a key driver of T1D pathogenic T cells. SOCS1 is capable of protecting against viral-mediated induction of T1D through the suppression of IFN-γ (45). Similarly, reduction of SOCS1 expression can lead to the expansion of pathogenic T cells in circulation (46).

Little is known about the function of RMI2 in T cells. However, a decrease in *RMI2* expression in other systems was shown to lead to the downregulation of RUNX2 (47), which is an important repressor of Tfh-cell differentiation (42). Skewing in the population of circulating Tfh cells or their precursors is highly associated with T1D (48). It is feasible that the downregulation of *RMI2* via the rs9746695 T1D enhancer risk allele leads to increased Tfh-cell formation.

The rs2358995 (chr1:114429515, A/C) *BCL2L15* intronic enhancer variant is an eQTL for both *BCL2L15* and *PTPN22.* We tested the effect of CRISPRi on expression by targeting the enhancers but did not see a significant change in *PTPN22* expression. Even though the enhancer forms a long-range interaction with the *PTPN22* gene body, there is no interaction with the gene promoter.

We validated an impact on *BCL2L15* expression. The *BCL2L15* gene is a proapoptotic member of the BCL2 family, which could impact T-cell proliferation and differentiation. It is not well studied, but a recent study showed that the knockdown of *BCL2L15* leads to the downregulation of STAT1 and STAT3 (49), which, if translated to CD4+ T cells, would have important implications for their function and differentiation.

*PTPN22* is strongly associated with type 1 diabetes, systemic lupus erythematosus, and rheumatoid arthritis. A coding variant within this gene was first linked to T1D and may even be causal (50, 51). It is noted as one of the most important non-HLA genetic risk factors in the predisposition to multiple autoimmune diseases. The lymphoid-specific protein is a molecular adapter protein associated with the negative regulation of TCR signaling, which may result in the survival of autoreactive T cells (51). We showed that the *PTPN22* eQTL variant rs2358995 does indeed reduce enhancer activity. Reduced expression of *PTPN22* through this enhancer variant may also predispose individuals to T1D.

Additionally, we found that the candidate TFs whose binding is predicted to be altered at T1D-associated enhancer variants have known interactions and play a role in shared pathways. For example, TCF1, LEF1, and ESRRA play important roles in CD4+ T-cell proliferation and differentiation (52, 53). The relationships between the candidate TFs would explain how different variants can alter binding for distinct TFs but result in a common phenotype, T1D. Altering TF binding to *cis-*regulatory elements provides a mechanism for how T1D-associated variants alter enhancer activity.

In summary, using MPRA in activated primary human CD4+ T cells, we identified four functional T1D variants residing in intronic enhancers of *CLEC16A* and *BCL2L15* genes. We utilized 3D genomic and eQTL data to assign the putative target genes such as *SOCS1* and *CLEC16A* and validated enhancer activity and target genes for variants containing distal enhancers using CRISPRi in Jurkat T cells. The validation identifies target genes that are well-known factors in driving the proinflammatory state of pathogenic T cells that drive ß-cell destruction. Dysregulated T-cell signaling due to functional regulatory variants also has the potential to skew T-cell differentiation, another key contribution to the etiology of T1D. We only validated a limited set of target genes, and future studies in primary T cells, including the use of base editors, could provide additional insight. We further predicted the effect of enhancer variants on TF binding, which indicated that shared pathways or regulatory networks may be at play in T1D. Collectively, these data prioritize these variants as candidate causal variants for T1D.

## Data availability statement

The sequencing data generated by this study are available from the NCBI Short Read Archive (SRA) under accession PRJNA1120596.

## Ethics statement

Ethical approval was not required for the studies involving humans because we used cells from a commercial biorepository that collects cells under their own IRB: Precision for Medicine Institutional Review Board (IRB) https://www.precisionformedicine.com/specialty-lab-services/biospecimens/. The studies were conducted in accordance with the local legislation and institutional requirements. The human samples used in this study were acquired from a commercial biorepository under IRB oversight. Written informed consent to participate in this study was not required from the participants or the participants’ legal guardians/next of kin in accordance with the national legislation and the institutional requirements.

## Author contributions

AM: Data curation, Formal analysis, Investigation, Methodology, Visualization, Writing – original draft, Writing – review & editing. AJ: Data curation, Formal analysis, Investigation, Methodology, Writing – original draft, Writing – review & editing. EW: Writing – review & editing, Data curation, Formal analysis, Investigation. ND: Writing – review & editing, Data curation, Formal analysis. MG: Investigation, Writing – review & editing. DH: Writing – review & editing, Investigation. RDH: Conceptualization, Funding acquisition, Supervision, Writing – original draft, Writing – review & editing.
