# Supplementary material for: Identification of functional enhancer variants associated with type I diabetes in CD4+ T cells
Source: Front Immunol. 2024 Jun 14;15:1387253. doi: 10.3389/fimmu.2024.1387253 (PMC11211866; doi:10.3389/fimmu.2024.1387253)
Supplement: Supplementary file 1 [file Presentation_1.pdf]

SUPPLEMENTARY MATERIALS

Identification of functional enhancer variants associated with type I diabetes in CD4+ T cells

Arpit Mishra[1, †], Ajay Jajodia[1, †], Eryn Weston[1], Naresh Doni Jayavelu[1], Mariana Garcia[1], Daniel Hossack[1], R. David Hawkins[1,2,3,*]

Table of Contents

Supplementary Figure 1. Upstream and downstream targets of enhancer variants.

Supplementary Figure 2. CRISPRi validation of enhancer-target gene interactions for *DEXI.*

Supplementary Table 1. Enhancer SNP MPRA.

Supplementary Table 2. Predicted allelic TF binding to validated enhancer variants.

Supplementary Table 3. CRISPRi guide RNAs and Taqman probes.

Supplementary Figure 1

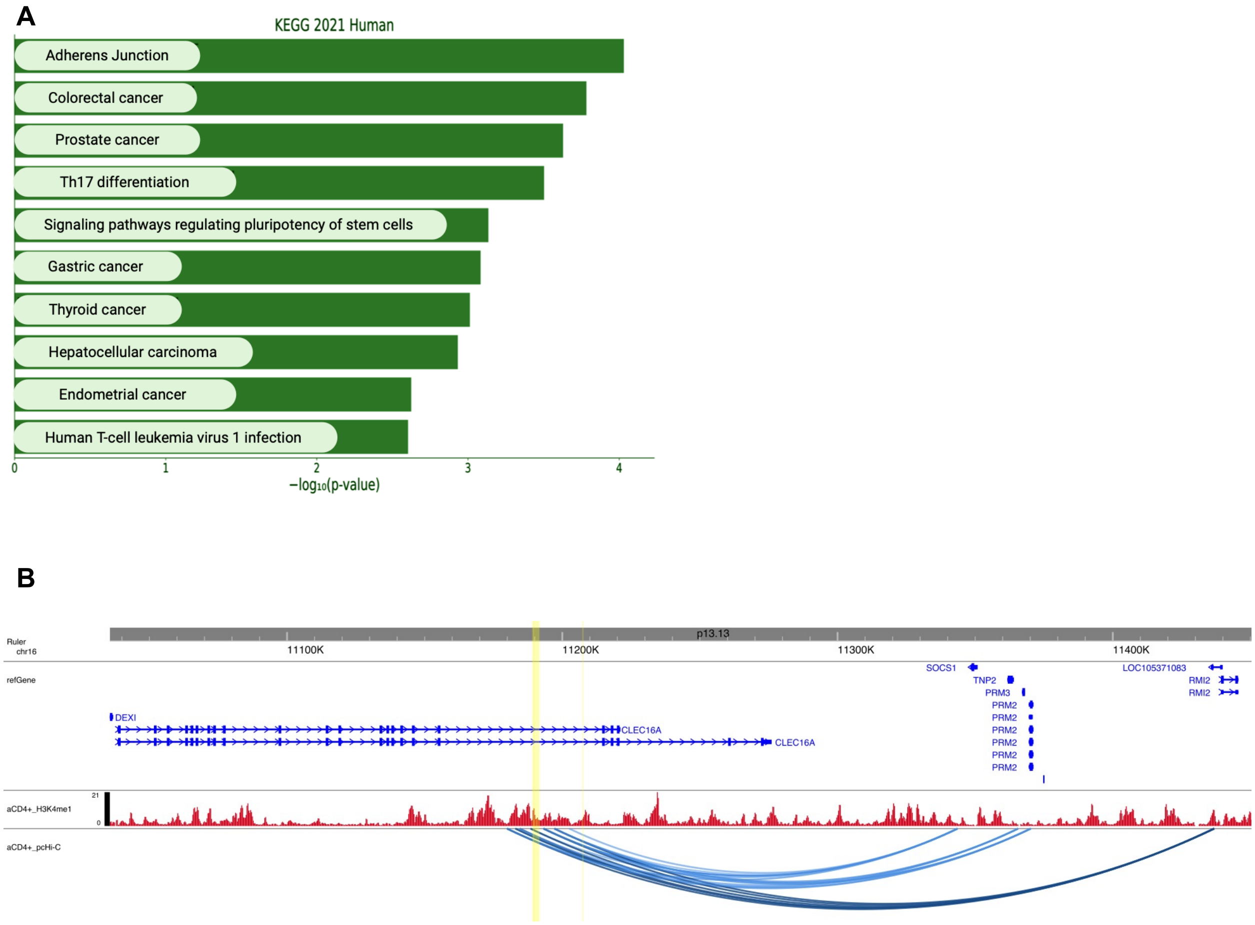

Supplementary Figure 1. Upstream and downstream targets of enhancer variants. **A.** KEGG pathways associated with T1D enhancer variant TFs. **B.** Promoter capture Hi-C enhancer target genes in the *CLEC16A* locus*.* Browser shot of *CLEC16A-RMI2* locus showing enhancer variants (yellow lines), H3K4me1 ChIP-seq data from activated CD4+ T cells (red) and activated CD4+ T cell pcHi-C data with enhancer variant interactions to target genes in blue (*SOCS1* gene) and dark gray (*RMI2* gene).

Supplementary Figure 2

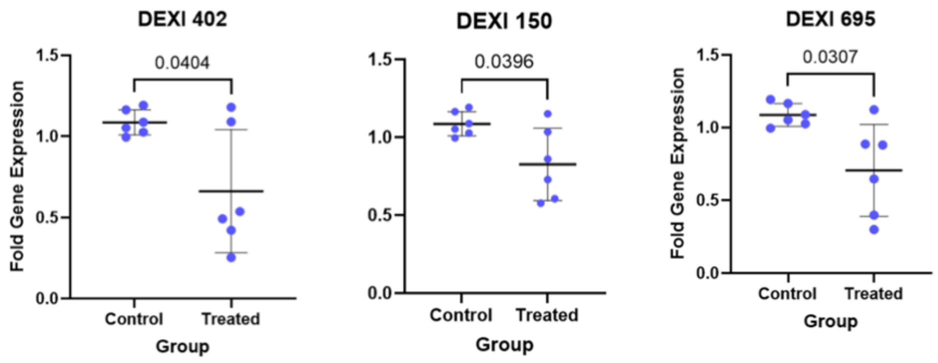

Supplementary Figure 2. CRISPRi validation of enhancer-target gene interactions for *DEXI* gene and enhancers harboring known eQTL variants rs12599402 (“402”), rs7203150 (“150”) and rs9746695 (“695”). Enhancer target gene expression level after enhancer (treated) repression by CRISPRi or using non-targeting control guide (Control). P values are indicated above the line in each comparison.
